# Decreased diastolic hydraulic forces incrementally associate with survival beyond conventional measures of diastolic dysfunction

**DOI:** 10.1038/s41598-023-41694-1

**Published:** 2023-09-29

**Authors:** Dhnanjay Soundappan, Angus S. Y. Fung, Daniel E. Loewenstein, David Playford, Geoffrey Strange, Rebecca Kozor, James Otton, Martin Ugander

**Affiliations:** 1 Kolling Institute, Royal North Shore Hospital, and University of Sydney, Sydney, Australia; 2 St Vincent’s Clinical School, University of New South Wales, Sydney, Australia; 3 Department of Clinical Physiology, Karolinska University Hospital, and Karolinska Institutet, Stockholm, Sweden; 4 School of Medicine, University of Notre Dame, Fremantle, Australia; 5 Faculty of Medicine and Health, University of Sydney, Sydney, Australia; 6 Department of Cardiology, Liverpool Hospital, University of New South Wales, Liverpool, Australia

**Keywords:** Physiology, Cardiology

## Abstract

Decreased hydraulic forces during diastole contribute to reduced left ventricular (LV) filling and heart failure with preserved ejection fraction. However, their association with diastolic function and patient outcomes are unknown. The aim of this retrospective, cross-sectional study was to determine the mechanistic association between diastolic hydraulic forces, estimated by echocardiography as the atrioventricular area difference (AVAD), and both diastolic function and survival. Patients (n = 5176, median [interquartile range] 5.5 [5.0–6.1] years follow-up, 1213 events) were selected from the National Echo Database Australia (NEDA) based on the presence of relevant transthoracic echocardiographic measures, LV ejection fraction (LVEF) ≥ 50%, heart rate 50–100 beats/minute, the absence of moderate or severe valvular disease, and no prior prosthetic valve surgery. NEDA contains echocardiographic and linked national death index mortality outcome data from 1985 to 2019. AVAD was calculated as the cross-sectional area difference between the LV and left atrium. LV diastolic dysfunction was graded according to 2016 guidelines. AVAD was weakly associated with E/e’, left atrial volume index, and LVEF (multivariable global R^2^ = 0.15, *p* < 0.001), and not associated with e’ and peak tricuspid regurgitation velocity. Decreased AVAD was independently associated with poorer survival, and demonstrated improved model discrimination after adjustment for diastolic function grading (C-statistic [95% confidence interval] 0.644 [0.629–0.660] vs 0.606 [0.592–0.621], *p* < 0.001) and E/e’ (0.649 [0.635–0.664] vs 0.634 [0.618–0.649], *p* < 0.001), respectively. Therefore, decreased hydraulic forces, estimated by AVAD, are weakly associated with diastolic dysfunction and demonstrate an incremental prognostic association with survival beyond conventional measures used to grade diastolic dysfunction.

## Introduction

Diastolic dysfunction contributes to the development of elevated left ventricular filling pressures and subsequent heart failure with preserved ejection fraction (HFpEF). Consequently, the physiological mechanisms underlying diastolic function remain an area of ongoing research in an attempt to develop effective therapeutic interventions for patients with HFpEF. Diastolic function is a composite description of the interaction between the mechanisms driving LV filling and the passive tension opposing LV filling which are responsible for lengthening the cardiomyocytes, increasing the volume of the LV, and generating the atrioventricular pressure gradient^1,2^. The term diastolic dysfunction can refer to mechanical abnormalities present during diastole which impair ventricular relaxation and filling^3^.

**Figure 1:**
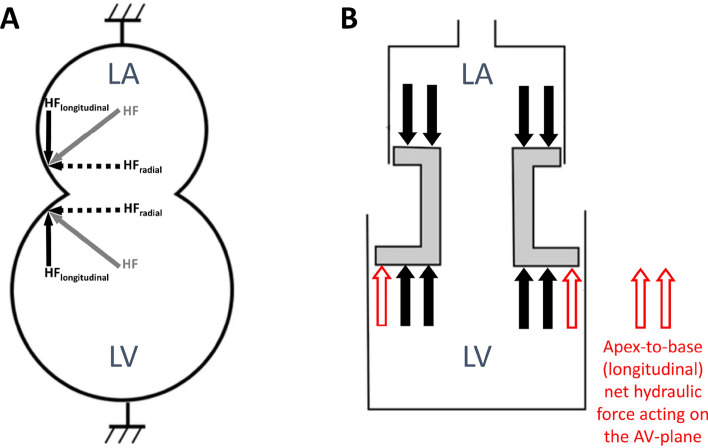
Schematic of hydraulic forces in the left atrium and ventricle. (**A**) During diastole, a hydraulic force (HF) is generated in the left atrium and ventricle perpendicular to the respective chamber walls, represented by the grey arrows in the left atrium and the left ventricle, respectively. These forces can be resolved into their longitudinal (HF_longitudinal_) and radial (HF_radial_) components as indicated by the solid black and dashed arrows, respectively. The radial component is counteracted by the pericardium and surrounding tissues whilst the longitudinal component represents the hydraulic force that contributes to the longitudinal motion of the atrioventricular plane during diastole. (**B**) Due to the larger surface area of the LV compared to the LA in a healthy individual, the LV generates a greater hydraulic force in the longitudinal direction, represented by the two additional red arrows. Abbreviations: AV = atrioventricular; HF = hydraulic force; HF_longitudinal_ = hydraulic force longitudinal component; HF_radial_ = hydraulic force radial component; LA = left atrium; LV = left ventricle. Figure adapted from^4^.

Hydraulic forces have recently been identified as a mechanism contributing to LV diastolic function^4^. Hydraulic force is calculated as the product of the pressure in a liquid, and the surface area with which that liquid is in contact, in accordance with Pascal’s Law^5^. Since the blood pressure in both chambers of the left heart is nearly identical during diastole^6^, the difference in cross-sectional short-axis area between the LV and left atrium (LA) provides the geometric basis for a net hydraulic force exuded on the atrioventricular plane in the apex-to-base direction (Fig. 1). This difference is termed the atrioventricular area difference (AVAD), and can be used as a surrogate measure of net hydraulic force.

Cardiac anatomy in healthy volunteers has already been shown to demonstrate a net hydraulic force directed towards the LA contributing to LV filling during most of diastole^4^. This force is expected to contribute to the diastolic longitudinal motion of the atrioventricular plane, facilitating LV lengthening and accounting for between 10 and 60% of the peak driving force of LV filling in healthy subjects^4^. Applying these findings to a clinical population, a recent study investigating changes to diastolic hydraulic forces in patients with pathological cardiac remodelling found that patients with HFpEF had a smaller AVAD compared to healthy controls throughout diastole, possibly limiting LV filling and contributing to the diastolic dysfunction^7^.

Although hydraulic forces have been demonstrated to be a physiological mechanism contributing to diastolic filling in healthy and diseased states, their association with diastolic function and patient outcomes remains unknown. Furthermore, if a decrease in hydraulic force is independently associated with survival, this may be a potential therapeutic target in HFpEF. Specifically, established methods for LA reduction surgery may provide an opportunity to decrease LA size relative to LV size, in an attempt to aid the contribution of hydraulic forces to LV filling. Therefore, the aim of this study was to determine the mechanistic association between diastolic hydraulic forces and both diastolic function and survival. We hypothesised that decreased diastolic hydraulic forces would be associated with diastolic dysfunction and poorer survival.

## Methods

### Study design

**Figure 2:**
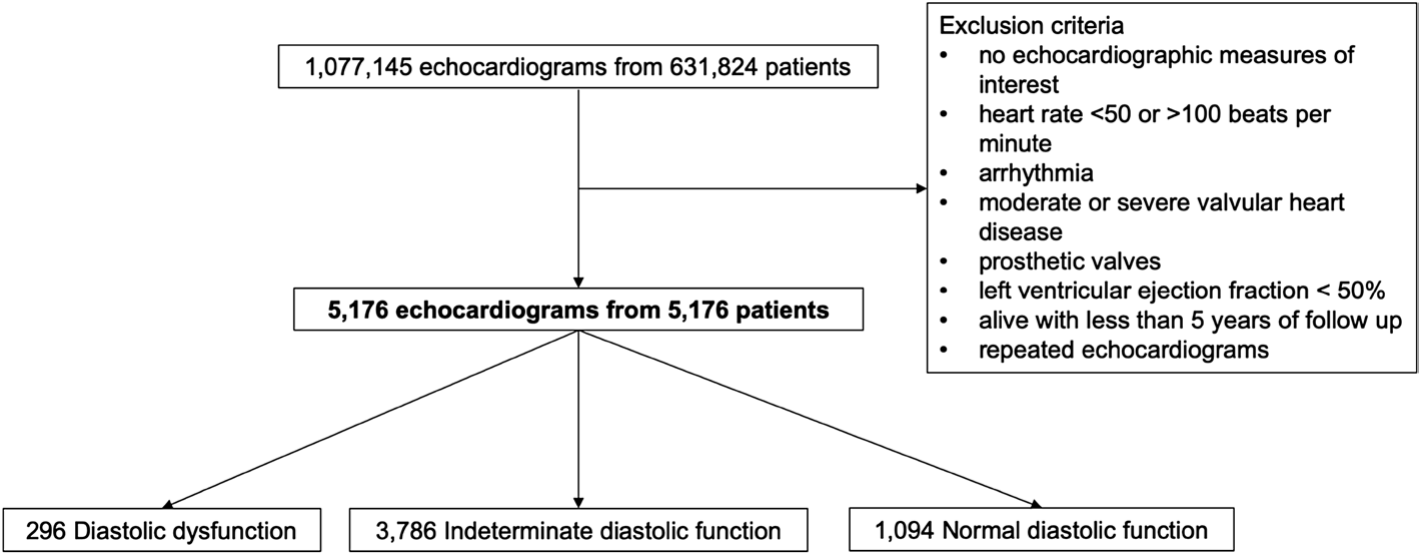
Patient inclusion flowchart. This flowchart describes the exclusion criteria of the study cohort.

The study population was derived using data from the National Echo Database Australia (NEDA). NEDA is an observational registry comprising of retrospectively and prospectively collected digital transthoracic echocardiographic measurements from patients referred to laboratories across Australia since 1985, and is linked to health outcome data, with patients followed up until the 2019 census or death^8^. Subjects were included following either a retrospective waiver of individual informed consent, or the absence of a prospective decision to opt out of enrolling in the study. The study complies with the Declaration of Helsinki, and the study and its design, including the retrospective waiver of consent, was approved by the lead ethics committee, namely the Ethics and Governance Office at the Royal Prince Alfred Hospital, Camperdown, Sydney, Australia (approval number: 2019/ETH06989), as well as the human research ethics committees of each participating site across Australia, respectively. Echocardiographic measurements from studies performed in laboratories adhering to clinical calibration standards were transferred to a central database, with quality control measures implemented to remove duplicate, inconsistent and physiologically impossible measurements^8^. The inclusion criteria for the study cohort were the presence of relevant echocardiographic measures, a LV ejection fraction (LVEF) greater than or equal to 50%, sinus rhythm, and a heart rate between 50 and 100 beats per minute. Echocardiographic measures of interest included LV end-diastolic diameter, LA end-systolic diameter, and at least two measures of diastolic function [E to septal e’ velocity ratio (E/e’), septal e’ velocity (e’ velocity), LA volume index (LAVI), or tricuspid regurgitation peak velocity]. These measures were selected to identify diastolic dysfunction as they are the recommended variables in the 2016 American Society of Echocardiography (ASE) and the European Association of Cardiovascular Imaging (EACVI) guidelines for evaluating LV diastolic function in patients with a LVEF greater than or equal to 50%^9^. Since average E/e’ was not consistently recorded in the database, E/septal e’ > 15 was used in the current study as per guideline recommendations. Exclusion criteria included moderate or severe valvular heart disease, and prosthetic valves. If multiple echocardiograms were available for a single patient, only the earliest recorded echocardiogram was included. The patient inclusion flowchart is described in Fig. 2. The current study is a mechanistic study with a large sample size based on clinically acquired data from a retrospective clinical database with objective outcome measures. Consequently, observers were blinded to the measurement of interest and clinical outcomes given that the calculation of AVAD was not postulated, nor were outcomes known, at the time of clinical measurement.

### Image analysis

**Figure 3:**
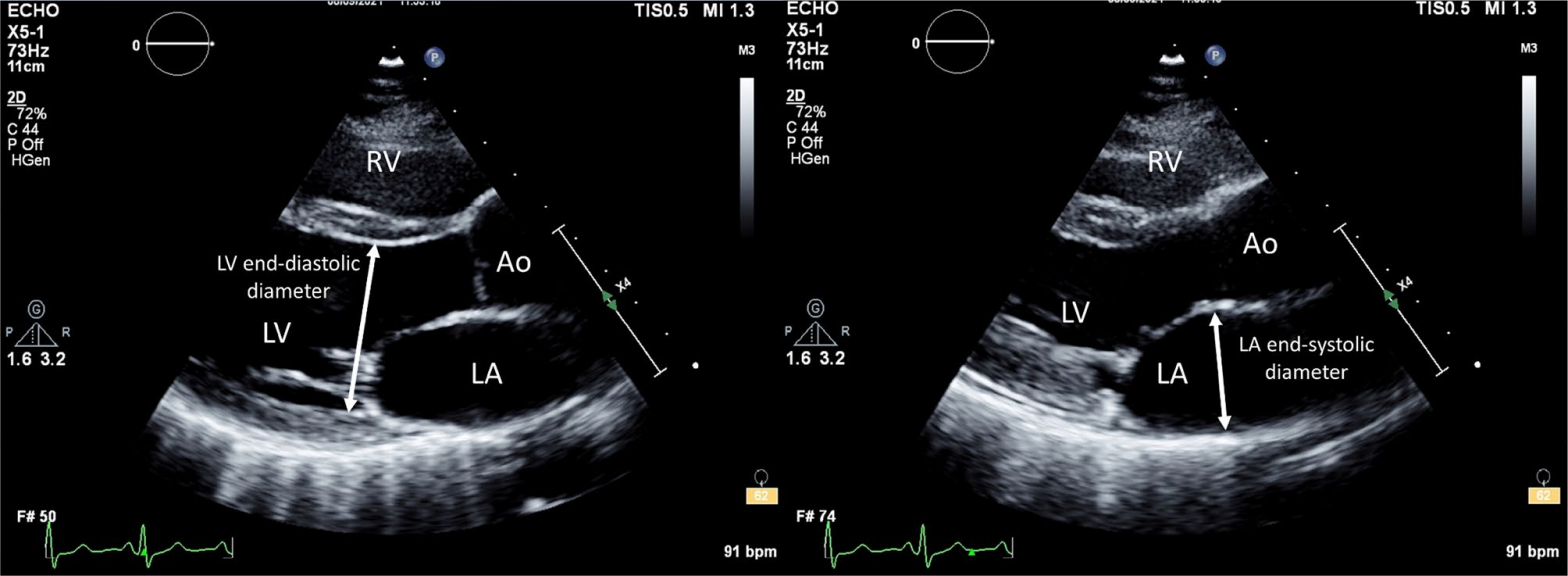
Measurement of atrioventricular area difference in transthoracic echocardiography. In routine echocardiography, atrioventricular area difference can be calculated as the difference between left ventricular end-diastolic short-axis cross-sectional area and left atrial end-systolic short-axis cross-sectional area. Short-axis cross-sectional area is calculated by circular approximation using left ventricular end-diastolic diameter and left atrial end-systolic diameter measured in a parasternal view. The atrial short-axis and ventricular short-axis areas represent the surface areas which contribute to the generation of hydraulic forces which contribute to the diastolic longitudinal motion of the atrioventricular plane. Abbreviations: Ao = aorta; LA = left atrium; LV = left ventricle; RV = right ventricle.

AVAD was calculated as the difference between LV and LA cross-sectional areas. LV and LA cross-sectional area were calculated by circular approximation using LV end-diastolic diameter and LA end-systolic diameter, respectively (Fig. 3).

For all measures, the designation of end diastole and end systole refers to LV systole and diastole. Using any available measures of diastolic function, patients were graded using the 2016 ASE/EACVI guidelines^9^. Specifically, patients were graded as having normal diastolic function if less than half of the available parameters met cut off values, indeterminate diastolic function if exactly half met cut off values, and diastolic dysfunction if more than half met cut off values.

### Statistical analysis

All statistical analyses were conducted using programming language, R (R Core Team, R Foundation for Statistical Computing, Vienna, Austria). The characteristics of the study cohort were calculated and reported as the median [interquartile range]. Differences in measured variables between patients were assessed using Wilcoxon rank-sum test. Univariable and multivariable linear regression were used to determine the relationship between AVAD and parameters of diastolic function in patient subgroups based on the availability of diastolic function measures. R^2^ values were used to represent the proportion of the variation in AVAD explained by the variables, whilst standardised beta coefficients were used to compare the strength of association with AVAD between variables. Survival analysis was conducted to examine the association between selected variables and time to death using Kaplan–Meier curves and Cox proportional hazards models. The end-points used were 5-year all-cause mortality and 5-year cardiovascular mortality. Univariable Cox regression models were used to evaluate the association between survival and AVAD, E/e’, and diastolic function grading, in the overall population and both sexes. Multivariable Cox regression models were used to evaluate the association between AVAD and survival, adjusting for conventional parameters of diastolic function. Finally, multivariable analyses were repeated in patient subgroups stratified by clinically relevant LVEF ranges. The proportional hazards assumption was confirmed by visual inspection of the Schoenfeld residuals, and by demonstrating a non-significant relationship between the residuals and time. Wald’s chi-square values were reported to compare the strength of association of different variables within regression models. Hazard ratios for continuous variables were scaled by standard deviation to allow for comparison between models, whilst hazard ratios for diastolic function grading were reported using normal diastolic function as a reference level. The goodness of fit of univariable and multivariable models was compared using the concordance (C) statistic, and differences in C-statistics were evaluated using a one-shot nonparametric approach independent of any overlap between the 95% confidence intervals of the two C-statistics being compared^10^. A *p*-value less than 0.05 was considered statistically significant.

## Results

### Study population

The population considered for this study consisted of 5176 patients, with cohort characteristics presented in Table 1. Patients were followed up for 5.5 [5.0–6.1] years, with 1213 deaths occurring over this period. All measures of diastolic function were consistent with better diastolic function in patients with an AVAD above the median, however not by a clinically meaningful magnitude (*p* < 0.001 for all).

**Table 1:** Baseline characteristics of study cohort.

Variable	Above median AVAD n = 2591 (50%)	Below median AVAD n = 2585 (50%)	Total population n = 5176 (100%)
Patient characteristics			
Gender, males (%)	1433 (55)	1054 (41)	2487 (48)
Age, years	56 [42–68]	67 [56–77]	62 [48–73]
Height, cm	170 [163–178]	165 [158–172]	168 [160–175]
Body Weight, kg	80 [69–94]	76 [65–90]	79 [67–91]
BMI, kg/m^2^	27.4 [24.0–31.6]	27.6 [24.2–31.9]	27.5 [24.1–31.7]
BSA, m^2^	2.0 [1.8–2.1]	1.9 [1.7–2.1]	1.9 [1.7–2.1]
Heart Rate, beats per minute	70 [65–79]	70 [60–80]	70 [62–80]
Systolic blood pressure, mmHg	126 [114–132]	129 [124–130]	128 [123–130]
Diastolic blood pressure, mmHg	80 [70–89]	78 [72–80]	78 [72–83]
Echocardiographic data			
E/A ratio	1.1 [0.8–1.3]	0.9 [0.7–1.2]	1.0 [0.8–1.3]
E wave velocity, cm/s	77 [62–92]	77 [62–91]	77 [62–91]
A wave velocity, cm/s	72 [59–88]	84 [68–101]	78 [63–95]
E/ e’ ratio	9.5 [7.4–11.7]	11.1 [8.8–14.2]	10.2 [7.9–13.0]
Septal e’, cm/s	8.0 [6.3–9.9]	6.6 [5.3–8.2]	7.2 [5.8–9.0]
LAVI, ml/m^2^	32 [27–38]	34 [28–43]	33 [27–40]
LVMI, g/m^2^	83 [71–99]	80 [67–97]	82 [69–98]
IVS diastolic wall thickness, mm	9.8 [8.6–11.0]	10.9 [9.5–12.0]	10.1 [9.0–11.7]
LVEF, %	64 [60–68]	65 [61–70]	65 [60–69]
TR peak velocity, m/s	2.4 [2.1–2.6]	2.5 [2.3–2.8]	2.4 [2.2–2.7]
LV EDD, cm	4.8 [4.5–5.2]	4.3 [4.0–4.6]	4.6 [4.2–5.0]
LA ESD, cm	3.3 [3.0–3.7]	3.8 [3.4–4.2]	3.5 [3.1–4.0]
AVAD, cm^2^	9.2 [7.7–11.3]	3.8 [1.8–5.2]	6.5 [3.8–9.2]

Data are reported as median [interquartile range]. Body surface area was calculated using the Mosteller formula. When comparing patients above and below the median population AVAD, all measures of diastolic function were consistent with better diastolic function in patients with an AVAD above the median (*p* < 0.001 for all), however not necessarily by a clinically meaningful magnitude.

*AVAD* atrioventricular area difference, *BMI* body mass index, *BP* blood pressure, *BSA* body surface area, *E/e’ ratio* E to septal e’ velocity ratio, *IVS Diastolic Wall Thickness* interventricular septum diastolic wall thickness, *LA ESD* left atrial end-systolic diameter, *LAVI* left atrial volume index, *LV EDD* left ventricular end-diastolic diameter, *LVEF* left ventricular ejection fraction, *LVMI* left ventricular mass index, *TR peak velocity* tricuspid regurgitation peak velocity.

### Atrioventricular area difference and diastolic function

AVAD was univariably associated with E/e’, e’ velocity, LAVI, peak tricuspid regurgitation velocity and LVEF. In multivariable linear regression, only E/e’, LAVI and LVEF remained associated with AVAD, with an overall weak association (global adjusted R^2^ = 0.15, *p* < 0.001, Table 2).

**Table 2:** Transthoracic echocardiographic measures of diastolic function and their association with atrioventricular area difference.

Measure	Univariable		Multivariable (319 patients)			
	R^2^	*p* value	Standardised $\beta$	*p* value	Global Adjusted R^2^	*p* value
E/e’ ratio (5176 patients)	0.06	< 0.001	− 0.31	< 0.001	0.15	< 0.001
e’ velocity, cm/s (4747 patients)	0.05	< 0.001	− 0.05	0.41		
LA volume index, cm^3^/m^2^ (957 patients)	0.03	< 0.001	− 0.12	0.04		
TR peak velocity, cm/s (2731 patients)	0.05	< 0.001	− 0.02	0.71		
LVEF, % (5176 patients)	0.03	< 0.001	− 0.19	< 0.001		

R^2^ values represent the proportion of the variation in AVAD explained by the variables, whilst standardised beta coefficients express the strength of the effect of each independent variable on AVAD. The number of patients included in each respective analysis is included in brackets.

*AVAD* atrioventricular area difference, *E/e’* E to septal e’ velocity ratio, *e' velocity* septal e' velocity, *LA volume index* left atrial volume index, *LVEF* left ventricular ejection fraction, *TR peak velocity* tricuspid regurgitation peak velocity.

### Prognostic value of atrioventricular area difference

**Figure 4:**
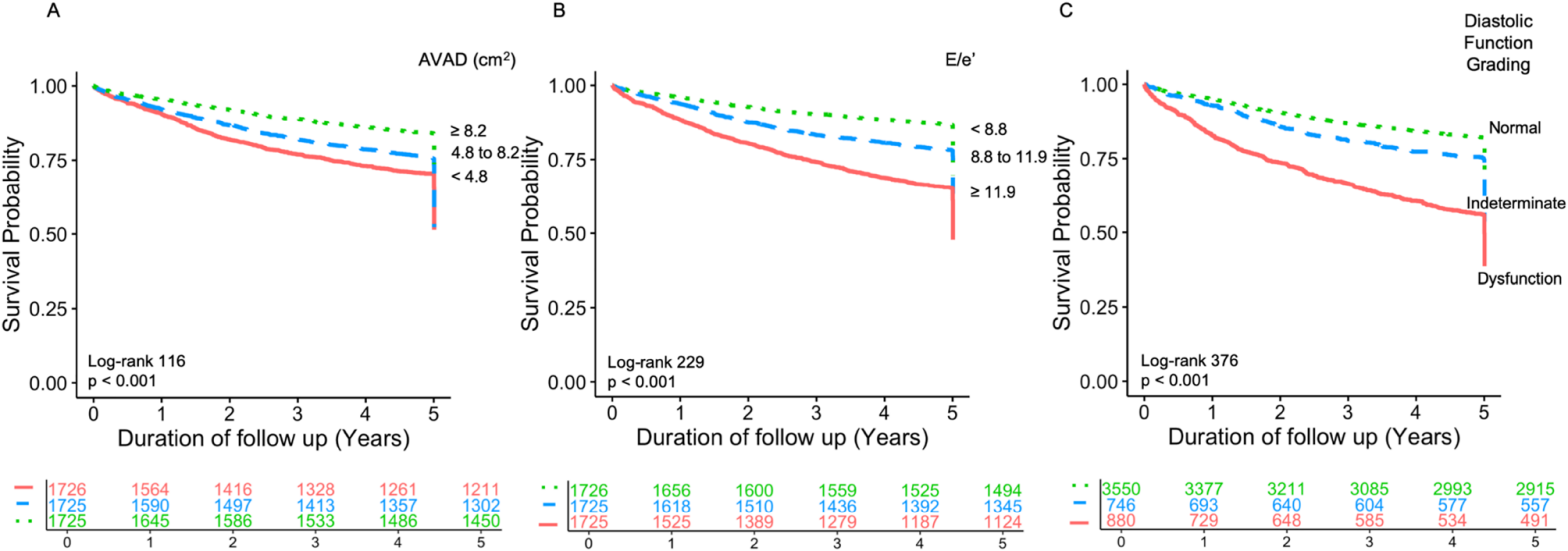
Survival curves for atrioventricular area difference, E/e’ ratio and diastolic function grading. Kaplan–Meier survival curves for (**A**) AVAD, (**B**) E/e’ and (**C**) diastolic function grading. Each curve is stratified by tertiles, with the respective risk table below each panel. Abbreviations: AVAD = atrioventricular area difference.

The results of univariable and multivariable Cox regression analyses using 5-year all-cause mortality as the outcome measure are summarised in Tables 3 and 4. In univariable Cox regression, AVAD was positively associated with survival (hazard ratio (HR) [95% confidence interval (CI)] 1.33 [1.26–1.40] per SD decrement, *p* < 0.001, Table 3) and E/e’ was negatively associated with survival (HR [95% CI] 1.49 [1.43–1.56] per SD increment, *p* < 0.001, Table 4). Both diastolic dysfunction and indeterminate diastolic function were associated with poorer survival compared to normal diastolic function (HR [95% CI] 2.99 [2.63–3.39], *p* < 0.001 for diastolic dysfunction). These results were replicated in sex-disaggregated analysis (data not shown). The Kaplan–Meier survival curves for these univariable analyses are represented in Fig. 4. In multivariable analyses, AVAD demonstrated improved model discrimination when included with diastolic function grading (C-statistic [95% CI] 0.644 [0.629–0.660] vs 0.606 [0.592–0.621], *p* < 0.001, Table 3) and E/e’ (C-statistic [95% CI] 0.649 [0.635–0.664] vs 0.634 [0.618–0.649], *p* < 0.001, Table 4) respectively. These results were unchanged when 5-year cardiovascular mortality was used as the outcome measure (Supplemental Tables 1 and 2). Additionally, a similar trend in results was observed when patients were stratified into subgroups of 50% ≤ LVEF < 60%, 60% ≤ LVEF < 75% and 75% ≤ LVEF (Supplemental Tables 3 and 4).

**Table 3:** Atrioventricular area difference and diastolic function grading as predictors of 5-year all-cause mortality.

LVEF ≥ 50% n = 5176, 1213 events 5.5 [5.0–6.1] yrs follow up	Univariable model				Multivariable model			
Variable	Chi-Square	HR [95% CI]	*p* value	C-statistic [95% CI]	Chi-Square	HR [95% CI]	*p* value	C-statistic [95% CI]
Diastolic dysfunction	288	2.99 [2.63–3.39]	< 0.001	0.606 [0.592–0.621]	208	2.64 [2.32–3.02]	< 0.001	0.644 [0.629–0.660]
Indeterminate diastolic function	22	1.48 [1.26–1.74]	< 0.001		17	1.42 [1.20–1.67]	< 0.001	
AVAD	111	1.33 [1.26–1.40]	< 0.001	0.588 [0.573–0.604]	41	1.20 [1.14–1.27]	< 0.001	

Cox regression was used to evaluate AVAD and diastolic function grading as predictors of 5-year all-cause mortality. Diastolic function grading was determined using the 2016 ASE/EACVI guidelines. Wald’s chi-square values were used to compare the strength of association of different variables within regression models. The hazard ratios for diastolic dysfunction and indeterminate diastolic function are reported with normal diastolic function as a reference level. The hazard ratio for AVAD is scaled by standard deviation decrement. The C-statistic of univariable and multivariable models was compared to evaluate differences in model discrimination.

*ASE* American Society of Echocardiography, *AVAD* atrioventricular area difference, *CI* confidence interval, *EACVI* European Association of Cardiovascular Imaging, *HR* hazard ratio, *LVEF* left ventricular ejection fraction.

**Table 4:** Atrioventricular area difference and E/e’ as predictors of 5-year all-cause mortality.

LVEF ≥ 50% n = 5176, 1213 events 5.5 [5.0–6.1] yrs follow up	Univariable model				Multivariable model			
Variable	Chi-Square	HR [95% CI]	*p* value	C-statistic [95% CI]	Chi-Square	HR [95% CI]	*p* value	C-statistic [95% CI]
E/e’ ratio	319	1.49 [1.43–1.56]	< 0.001	0.634 [0.618–0.649]	225	1.43 [1.36–1.49]	< 0.001	0.649 [0.635–0.664]
AVAD	111	1.33 [1.26–1.40]	< 0.001	0.588 [0.573–0.604]	42	1.21 [1.14–1.28]	< 0.001	

Cox regression was used to evaluate AVAD and E/e’ as predictors of 5-year all-cause mortality. Wald’s chi-square values were used to compare the strength of association of different variables within regression models. The hazard ratio for E/e’ ratio is scaled by standard deviation increment, and AVAD by standard deviation decrement. The C-statistic of univariable and multivariable models was compared to evaluate differences in model discrimination.

*AVAD* atrioventricular area difference, *CI* confidence interval, *E/e’* E to septal e’ velocity ratio, *HR* hazard ratio, *LVEF* left ventricular ejection fraction.

## Discussion

The main finding of this study is that decreased diastolic hydraulic forces, estimated by AVAD, are associated with poor survival. This association between diastolic hydraulic forces and survival is independent of, and incremental to, current measures of diastolic function. Taken together, this suggests that further investigation evaluating increased LA size relative to LV size as a potential therapeutic target in HFpEF is justified.

Expanding on previous attempts to find predictors of diastolic dysfunction^11,12^, diastolic hydraulic forces provide a macroscopic explanation to a part of the physiology behind diastolic function, thus incorporating the effects of blood pressure and cardiac geometry into a unified theoretical framework. It is important to note that diastolic dysfunction refers to impaired myocardial relaxation and increased chamber stiffness contributing to raised LV filling pressures which is one of the mechanisms underlying the development of HFpEF. The contribution of diastolic hydraulic forces to LV filling in health has been found to be of comparable magnitude to other diastolic mechanisms such as active relaxation and restoring forces^4^. Meanwhile, conventional parameters recommended by the ASE/EACVI to grade diastolic function account for the contribution of LV early diastolic recoil, LV relaxation, LV filling pressure, and LA pressure^13^. The recommended algorithm from these guidelines has been previously associated with increased cardiovascular-related and all-cause mortality^14^. The current study shows that AVAD provides prognostic value beyond these measures to predict survival in a clinical population. When compared directly with E/e’, the echocardiographic parameter most closely related to LV filling pressure^15,16^, AVAD retained an independent association with survival, albeit with a somewhat weaker strength of association. These results, including the finding of merely a weak association between AVAD and conventional diastolic measures, indicate that AVAD provides novel information when assessing diastolic function and prognosis in patients.

### Method for calculating AVAD

Our study calculated AVAD as the difference between approximated LV end-diastolic and LA end-systolic short-axis cross-sectional areas, respectively. Short-axis cross-sectional area was measured as it represents the two-dimensional surface area upon which hydraulic forces in the base-apex direction act on the mitral annulus during diastole. By comparison, while the third dimension of chamber length in the base-apex direction is essential for quantifying chamber volume, it does not influence the hydraulic forces in the base-apex direction that are acting upon the mitral annulus. Routine echocardiographic measurements of the LV are performed at end systole and end diastole, while the dimensions of the LA are measured only at end systole. However, the contribution of AVAD to the net diastolic hydraulic force is most accurately calculated during mid-diastasis when LA and LV pressures equalise^4^. Whilst there is no meaningful magnitude of change in LV cross-sectional area between mid-diastasis and end diastole, LA cross-sectional area is greater at end systole than at mid diastasis^4^. Specifically, in a study of healthy volunteers, LA short-axis area increased from 14 to 19 cm^2^ between mid-diastasis and end systole^4^. This change in area is related to the movement of the mitral annular plane, or mitral annular plane systolic excursion (MAPSE)^17,18^. Specifically, as the mitral annular plane is displaced towards the apex during systole, LA cross-sectional area increases. To account for the potential influence of MAPSE on our measurement of AVAD, we repeated our statistical analyses in patients stratified by subgroups of LVEF above 50%, and observed a similar trend in results. It is well established that MAPSE is positively associated with LVEF^19–22^. Therefore, it can be inferred that patients with a similar LVEF also have an adequately similar MAPSE and the movement of the mitral annular plane does not considerably affect the differences in AVAD between these patients. This is supported by the negligibly weak association found between AVAD and LVEF within each subgroup of LVEF above 50% (R^2^ = 0.01–0.03, *p* < 0.001, Supplemental Table 5). As such, when comparing patients with similar LVEF, AVAD measured using LV end-diastolic diameter and LA end-systolic diameter can be used as a reasonable surrogate for diastolic hydraulic forces that ideally would be based on AVAD measured during mid-diastasis.

### The relative geometry of the left ventricle and left atrium

Cardiac geometry is known to play an integral role in cardiovascular health. Changes to the structure of the LV and LA, either independently or simultaneously, have been demonstrated to impair function and contribute to cardiovascular disease and outcomes^23–29^. Whilst LA and LV measures are often studied separately, it has been suggested that a single parameter accounting for the complex interplay between LA and LV physiology may be a more clinically useful predictor of cardiovascular disease and survival^30^. Extending on these ideas, it has recently been demonstrated that a left atrioventricular coupling index (LACI) was a strong predictor of cardiovascular events and death, with improved discrimination and reclassification power when compared to individual LA and LV measures^30^. LACI was calculated by dividing LA end-diastolic volume by LV end-diastolic volume, with the authors acknowledging that LA and LV function and pressure are most closely related during diastole. Considering that cardiac chamber volume is dependent on mean cross-sectional area, the findings using LACI represent an approximate surrogate measure of diastolic hydraulic forces, albeit without specifically measuring the difference in LV and LA short-axis cross-sectional areas.

### The potential of left atrial reduction surgery

The independent prognostic value of AVAD indicates that increased LA size relative to LV size may be a new therapeutic target in patients with HFpEF. In HFpEF, elevated LV filling pressures are propagated to the LA, resulting in remodelling and dilatation^24,29,31^. This may result in the LA cross-sectional area becoming equal to or larger than the LV cross-sectional area, orienting the net diastolic hydraulic force towards the apex of the heart and opposing LV filling^7^. Decreasing LA size in these patients may potentially restore the contribution of diastolic hydraulic forces to LV filling, possibly improving both diastolic function and survival. LA reduction surgery is already an established procedure^32–36^ primarily indicated alongside other surgical procedures to treat an enlarged LA or chronic atrial fibrillation in patients with mitral valve disease^33^. Several studies have highlighted the benefits of LA reduction surgery, most commonly resulting in increased restoration and maintenance of sinus rhythm, with no significant increases in mortality or post-operative complications^37–43^. The mechanism for these improvements is not clearly understood. However, it is thought to be related to electrophysiological remodelling and increased wall stress^44,45^. We hypothesise that these benefits may also be attributed to the subsequent improvement in net diastolic hydraulic force and diastolic function. Diastolic dysfunction is thought to contribute to increased atrial afterload, stretching and wall stress, ultimately increasing the risk of atrial fibrillation^46,47^. Considering the importance of cardiac geometry and diastolic function in HFpEF as well, the current results support the notion that LA reduction surgery concomitant with otherwise indicated open-heart surgery is a treatment option that merits prospective evaluation with regards to improving symptoms and outcomes in carefully selected patients with HFpEF.

### Limitations

As it currently stands, NEDA does not contain clinical data relating to patient comorbidities, cardiovascular disease risk factors, and pharmacotherapy. This information may impact results, as these clinical details affect both survival and diastolic function, especially since the NEDA population primarily consists of patients referred for echocardiography due to known or suspected cardiovascular disease. Additionally, the retrospective registry nature of the NEDA data used in the current study precludes analysis of individual laboratory reproducibility and assessment of inter- and intra-observer reliability of measurements. Nevertheless, echocardiographic data paired with survival from NEDA has previously been used to draw important prognostic information on pulmonary hypertension, aortic stenosis, and diastolic dysfunction, reflecting the validity and reliability of NEDA data, as well as the robustness of using mortality end-points despite a lack of clinical data^14,48–51^. Inspection of descriptive statistics of the included measures (data not shown) also did not indicate any reason for concern regarding systematic variations in the data. Additionally, our study may have been limited by the availability of data on the NEDA database. There was a limitation in the number of echocardiographic studies measuring both LA and LV diameter concurrently, thereby reducing the study population available for analysis. Measurement variability inherent to the assumption of circular cross-sectional areas and the variability in selecting the locations of measuring LV and LA diameters may have also limited our methodology. Alternative methods of measuring cardiac geometry, such as atrial and ventricular volumes using apical views to account for variations, were unavailable in the database. However, these alternatives were not needed for the mechanistic validation undertaken in the current study. In considering this mechanistic validation provided in the current study, it is important to consider the large proportion of the study graded as having indeterminate diastolic function. Ultimately, this represents the difficulty in stratifying the complex spectrum of diastolic function, acknowledged by the ASE/EACVI guidelines. Indeed, the guidelines encourage further testing with a wider variety of echocardiographic and haemodynamic measures in such instances. The fact that the current study consistently demonstrates a clear prognostic value using clinically acquired data despite these limitations indicates the robustness of these prognostic relationships. This also accounts for the magnitude of the predictive power of AVAD as well as the correlation with diastolic function markers, as the current study primarily aimed to demonstrate a potential mechanistic association between AVAD and both diastolic function and survival. Finally, our study did not perform a sample size calculation given that our study population was larger than any previously cited studies examining diastolic function and survival, and post-hoc power analyses are not appropriate for an already observed statistic and standard error. In light of the findings and limitations of the current study, future studies should look to further validate the prognostic value of AVAD, while overcoming the limitations of the current study by ideally measuring AVAD in mid diastole (diastasis), and including relevant clinical characteristics.

## Conclusions

A decrease in diastolic hydraulic forces, estimated by AVAD, is weakly associated with diastolic dysfunction, and independently associated with poorer survival in a clinical population referred for echocardiography. Increased LA size relative to the LV may impair the longitudinal motion of the atrioventricular plane during diastole, impairing LV filling and contributing to a poorer prognosis for patients with cardiovascular disease.

### Supplementary Information

Supplementary Tables.

## Data Availability

The data that support the findings of this study are available from the National Echo Database Australia but restrictions apply to the availability of these data, which were used under license for the current study, and so are not publicly available. Data are however available from the corresponding author (M.U.) upon reasonable request and with permission of the National Echo Database Australia.
