# Supplementary material for: Decreased diastolic hydraulic forces incrementally associate with survival beyond conventional measures of diastolic dysfunction
Source: Sci Rep. 2023 Sep 29;13:16396. doi: 10.1038/s41598-023-41694-1 (PMC10541860; doi:10.1038/s41598-023-41694-1)
Supplement: Supplementary file 1 — Supplementary Tables. [file 41598_2023_41694_MOESM1_ESM.docx]

**Decreased diastolic hydraulic forces incrementally associate with survival beyond conventional measures of diastolic dysfunction**

Dhnanjay Soundappan^a,b^, Angus SY Fung^a,b^, Daniel E Loewenstein MD^a,c^,
David Playford MBBS PhD^d^, Geoffrey Strange PhD^d,e^, Rebecca Kozor MBBS PhD^a^,
James Otton MBBS PhD^f^, *Martin Ugander MD PhD^a,b,c^

^a^Kolling Institute, Royal North Shore Hospital, and University of Sydney, Sydney, Australia

^b^St Vincent’s Clinical School, University of New South Wales, Sydney, Australia
^c^Department of Clinical Physiology, Karolinska University Hospital, and Karolinska Institutet, Stockholm, Sweden

^d^School of Medicine, University of Notre Dame, Fremantle, Australia
^e^Faculty of Medicine and Health, University of Sydney, Sydney, Australia
^f^Department of Cardiology, Liverpool Hospital, University of New South Wales, Liverpool, Australia

**Email addresses:**

Dhnanjay Soundappan: [dhnanjay.soundappan@gmail.com](mailto:dhnanjay.soundappan@gmail.com)
Angus SY Fung: [angussyfung@gmail.com](mailto:angussyfung@gmail.com)

Daniel E Loewenstein: [loewenstein.daniel@gmail.com](mailto:loewenstein.daniel@gmail.com)

David Playford: [david@playford.biz](mailto:david@playford.biz)

Geoffrey Strange: [geoff@mozaicsolutions.com.au](mailto:geoff@mozaicsolutions.com.au)

Rebecca Kozor: [rebeccakozor@gmail.com](mailto:rebeccakozor@gmail.com)

James Otton: [jotton@gmail.com](mailto:jotton@gmail.com)

*Martin Ugander: [martin.ugander@ki.se](mailto:martin.ugander@ki.se)

**Address for correspondence:**

Professor Martin Ugander, MD, PhD, FCSANZ
Kolling Building, Level 12, Royal North Shore Hospital
St Leonards, Sydney, NSW 2065, Australia
E-mail: martin.ugander@sydney.edu.au, mobile: +61481134220

**Supplemental Table 1. Atrioventricular area difference and diastolic function grading as predictors of 5-year cardiovascular mortality.**

| LVEF ≥ 50% n = 5176, 173 events  5.5 [5.0–6.1] yrs follow up | **Univariable Model** | | | | **Multivariable Model** | | | |
| --- | --- | --- | --- | --- | --- | --- | --- | --- |
| **Variable** | Chi-Square | HR [95% CI] | p value | C-statistic  [95% CI] | Chi-Square | HR [95% CI] | p value | C-statistic  [95% CI] |
| Diastolic Dysfunction | 111 | 5.80 [4.18 – 8.03] | <0.001 | 0.687  [0.648 – 0.726] | 84 | 4.94 [3.51 – 6.95] | <0.001 | 0.718  [0.680 – 0.757] |
| Indeterminate Diastolic Function | 10 | 2.02 [1.29 – 3.17] | 0.002 |  | 8 | 1.91 [1.22 – 2.99] | 0.005 |  |
| AVAD | 37 | 1.51 [1.32 – 1.73] | <0.001 | 0.620  [0.580 – 0.661] | 10 | 1.27 [1.10 – 1.46] | 0.001 |  |

Univariable and multivariable cox regression was used to evaluate AVAD and diastolic function grading as predictors of 5-year cardiovascular mortality. Diastolic function grading was determined using the 2016 ASE/EACVI guidelines. Wald’s chi-square values were used to compare the strength of association of different variables within regression models. The hazard ratios for diastolic dysfunction and indeterminate diastolic function are reported with normal diastolic function as a reference level. The hazard ratio for AVAD is scaled by standard deviation decrement. The C-statistic of univariable and multivariable models was compared to evaluate differences in model discrimination. Abbreviations: ASE = American Society of Echocardiography; AVAD = atrioventricular area difference; CI = confidence interval; EACVI = European Association of Cardiovascular Imaging; HR = hazard ratio; LVEF = left ventricular ejection fraction.

**Supplemental Table 2. Atrioventricular area difference and E/e’ as predictors of 5-year cardiovascular mortality.**

| LVEF ≥ 50% n = 5176, 1213 events  5.5 [5.0–6.1] yrs follow up | **Univariable Model** | | | | **Multivariable Model** | | | |
| --- | --- | --- | --- | --- | --- | --- | --- | --- |
| **Variable** | Chi-Square | HR [95% CI] | p value | C-statistic  [95% CI] | Chi-Square | HR [95% CI] | p value | C-statistic  [95% CI] |
| E/e’ ratio | 86 | 1.64 [1.48 – 1.82] | <0.001 | 0.675  [0.634 – 0.716] | 56 | 1.53 [1.37 – 1.71] | <0.001 | 0.699  [0.658 – 0.739] |
| AVAD | 37 | 1.51 [1.32 – 1.73] | <0.001 | 0.620 [0.580 – 0.661] | 16 | 1.35 [1.17 – 1.56] | <0.001 |  |

Univariable and multivariable cox regression was used to evaluate AVAD and E/e’ as predictors of 5-year cardiovascular mortality. Wald’s chi-square values were used to compare the strength of association of different variables within regression models. The hazard ratio for E/e’ ratio is scaled by standard deviation increment, and AVAD by standard deviation decrement. The C-statistic of univariable and multivariable models was compared to evaluate differences in model discrimination. Abbreviations: AVAD = atrioventricular area difference; CI = confidence interval; E/e’ = E to septal e’ velocity ratio; HR = hazard ratio; LVEF = left ventricular ejection fraction.

## **Supplemental Table 3. Atrioventricular area difference and diastolic function grading as predictors of 5-year all-cause mortality in left ventricular ejection fraction subgroups**

| 75% ≤ LVEF n = 296, 82 events  5.5 [3.8–8.0] yrs follow up | **Univariable Model** | | | | **Multivariable Model** | | | |
| --- | --- | --- | --- | --- | --- | --- | --- | --- |
| **Variable** | Chi-Square | HR [95% CI] | p value | C-statistic  [95% CI] | Chi-Square | HR [95% CI] | p value | C-statistic  [95% CI] |
| Diastolic Dysfunction | 5 | 1.73 [1.07 – 2.80] | 0.03 | 0.572  [0.516 – 0.628] | 2 | 1.47 [0.88-2.47] | 0.14 | 0.608  [0.547 – 0.669] |
| Indeterminate Diastolic Function | 1 | 0.74 [0.39 – 1.44] | 0.38 |  | 1 | 0.68 [0.35-1.32] | 0.26 |  |
| AVAD | 6 | 1.32 [1.05 – 1.66] | 0.02 | 0.588  [0.526 – 0.649] | 4 | 1.26 [0.99-1.61] | 0.06 |  |
| 60% ≤ LVEF < 75% n = 3786, 840 events  5.5 [5.1–6.0] yrs follow up | **Univariable Model** | | | | **Multivariable Model** | | | |
| **Variable** | Chi-Square | HR [95% CI] | p value | C-statistic | Chi-Square | HR [95% CI] | p value | C-statistic |
| Diastolic Dysfunction | 235 | 3.25 [2.80 – 3.78] | <0.001 | 0.614  [0.597 – 0.631] | 168 | 2.86 [2.44 – 3.35] | <0.001 | 0.655  [0.637 – 0.673] |
| Indeterminate Diastolic Function | 20 | 1.58 [1.30 – 1.93] | <0.001 |  | 17 | 1.52 [1.25 – 1.86] | <0.001 |  |
| AVAD | 84 | 1.36 [1.27 – 1.45] | <0.001 | 0.595  [0.576 – 0.614] | 26 | 1.20 [1.12 – 1.29] | <0.001 |  |
| 50% ≤ EF < 60% n = 1094, 291 events  5.5 [4.6–8.2] yrs follow up | **Univariable Model** | | | | **Multivariable Model** | | | |
| **Variable** | Chi-Square | HR [95% CI] | p value | C-statistic | Chi-Square | HR [95% CI] | p value | C-statistic |
| Diastolic Dysfunction | 51 | 2.62 [2.01 – 3.41] | <0.001 | 0.595  [0.564 – 0.625] | 37 | 2.33 [1.77 – 3.05] | <0.001 | 0.632  [0.601 – 0.664] |
| Indeterminate Diastolic Function | 4 | 1.38 [1.01 – 1.88] | 0.04 |  | 2 | 1.28 [0.94 – 1.75] | 0.12 |  |
| AVAD | 29 | 1.30 [1.18 – 1.43] | <0.001 | 0.586  [0.555 – 0.617] | 16 | 1.23 [1.11 – 1.35] | <0.001 |  |

Survival analyses were repeated in LVEF subgroups of 50% ≤ LVEF < 60%, 60% ≤ LVEF < 75% and 75% ≤ LVEF to account for the influence of mitral annular plane systolic excursion on our measurement of AVAD. A similar trend in results were observed in all groups. Diastolic function grading was determined using the 2016 ASE/EACVI guidelines. Wald’s chi-square values were used to compare the strength of association of different variables within regression models. The hazard ratios for diastolic dysfunction and indeterminate diastolic function are reported with normal diastolic function as a reference level. The hazard ratio for AVAD is scaled by standard deviation decrement. The C-statistic of univariable and multivariable models was compared to evaluate differences in model discrimination. Abbreviations: ASE = American Society of Echocardiography; AVAD = atrioventricular area difference; CI = confidence interval; EACVI = European Association of Cardiovascular Imaging; HR = hazard ratio; LVEF = left ventricular ejection fraction.

## **Supplemental Table 4. Atrioventricular area difference and E/e’ as predictors of 5-year all-cause mortality in left ventricular ejection fraction subgroups**

| 75% ≤ LVEF n = 296, 82 events  5.5 [3.8–8.0] yrs follow up | **Univariable Model** | | | | **Multivariable Model** | | | |
| --- | --- | --- | --- | --- | --- | --- | --- | --- |
| **Variable** | Chi-Square | HR [95% CI] | p value | C-statistic  [95% CI] | Chi-Square | HR [95% CI] | p value | C-statistic  [95% CI] |
| E/e’ ratio | 10 | 1.35 [1.12 – 1.62] | 0.001 | 0.588  [0.527 – 0.650] | 6 | 1.28 [1.05 – 1.55] | 0.01 | 0.609  [0.548 – 0.670] |
| AVAD | 6 | 1.32 [1.05 – 1.66] | 0.02 | 0.588  [0.526 – 0.649] | 2 | 1.20 [0.94 – 1.53] | 0.14 |  |
| 60% ≤ LVEF < 75% n = 3786, 840 events  5.5 [5.1–6.0] yrs follow up | **Univariable Model** | | | | **Multivariable Model** | | | |
| **Variable** | Chi-Square | HR [95% CI] | p value | C-statistic | Chi-Square | HR [95% CI] | p value | C-statistic |
| E/e’ ratio | 249 | 1.57 [1.48 – 1.65] | <0.001 | 0.641  [0.623 – 0.659] | 176 | 1.49 [1.40 – 1.58] | <0.001 | 0.657  [0.639 – 0.675] |
| AVAD | 84 | 1.36 [1.27 – 1.45] | <0.001 | 0.595  [0.576 – 0.614] | 31 | 1.23 [1.14 – 1.31] | <0.001 |  |
| 50% ≤ EF < 60% n = 1094, 291 events  5.5 [4.6–8.2] yrs follow up | **Univariable Model** | | | | **Multivariable Model** | | | |
| **Variable** | Chi-Square | HR [95% CI] | p value | C-statistic | Chi-Square | HR [95% CI] | p value | C-statistic |
| E/e’ ratio | 69 | 1.39 [1.28 – 1.50] | <0.001 | 0.633  [0.602 – 0.663] | 47 | 1.33 [1.22 – 1.44] | <0.001 | 0.652  [0.622 – 0.682] |
| AVAD | 29 | 1.30 [1.18 – 1.43] | <0.001 | 0.586  [0.555 – 0.617] | 12 | 1.21 [1.09 – 1.34] | <0.001 |  |

Survival analyses were repeated in LVEF subgroups of 50% ≤ LVEF < 60%, 60% ≤ LVEF < 75% and 75% ≤ LVEF to account for the influence of mitral annular plane systolic excursion on our measurement of AVAD. A similar trend in results were observed in all groups. Wald’s chi-square values were used to compare the strength of association of different variables within regression models. The hazard ratio for E/e’ ratio is scaled by standard deviation increment, and AVAD by standard deviation decrement. The C-statistic of univariable and multivariable models was compared to evaluate differences in model discrimination. Abbreviations: AVAD = atrioventricular area difference; CI = confidence interval; C-statistic = concordance statistic; E/e’ = E to septal e’ velocity ratio; HR = hazard ratio; LVEF = left ventricular ejection fraction.

## **Supplemental Table 5. Univariable association between left ventricular ejection fraction and atrioventricular area difference in left ventricular ejection fraction subgroups**

| **LVEF group** | **R^2^** | **p value** |
| --- | --- | --- |
| LVEF ≥ 75% | 0.01 | 0.03 |
| 60% ≤ LVEF < 75% | 0.01 | <0.001 |
| 50% ≤ LVEF < 60% | 0.01 | 0.001 |
| LVEF ≤ 50% | 0.03 | <0.001 |

Univariable linear regression was used to determine the association between AVAD and LVEF. Patients with a similar LVEF are known to have a comparable mitral annular plane systolic excursion, meaning the movement of the mitral annular plane does not meaningfully affect differences in AVAD between patients with a similar LVEF. Abbreviations: AVAD = atrioventricular area difference; LVEF = left ventricular ejection fraction.
